# A Sensitive and Rapid Method to Determine the Adhesion Capacity of Probiotics and Pathogenic Microorganisms to Human Gastrointestinal Mucins

**DOI:** 10.3390/microorganisms6020049

**Published:** 2018-05-29

**Authors:** Bélinda Ringot-Destrez, Zéa D’Alessandro, Jean-Marie Lacroix, Muriel Mercier-Bonin, Renaud Léonard, Catherine Robbe-Masselot

**Affiliations:** 1 Univ.lille, CNRS, UMR8576-UGSF-Unité de Glycobiologie Structurale et Fonctionnelle, F59000 Lille, France; belinda.ringot@gmail.com (B.R.-D.); zea.dalessandro@etudiant.univ-lille1.fr (Z.D.); Jean-marie.lacroix@univ-lille.fr (J.M.L.); renaud.leonard@univ-lille1.fr (R.L.); 2 Toxalim (Research Centre in Food Toxicology), Université de Toulouse, INRA, ENVT, INP-Purpan, UPS, 31000 Toulouse, France; muriel.mercier-bonin@inra.fr; 3 Unité de Glycobiologie Structurale et Fonctionnelle, Campus CNRS de la Haute Borne, 50 avenue de Halley, 59658 Villeneuve d’Ascq, France

**Keywords:** bacterial adhesion, mucin, *O*-glycosylation, sialic acids, probiotics, bacterial pathogens

## Abstract

Mucus is the habitat for the microorganisms, bacteria and yeast that form the commensal flora. Mucins, the main macromolecules of mucus, and more specifically, the glycans that cover them, play essential roles in microbial gastrointestinal colonization. Probiotics and pathogens must also colonize mucus to have lasting positive or deleterious effects. The question of which mucin-harboured glycan motifs favour the adhesion of specific microorganisms remains very poorly studied. In the current study, a simple test based on the detection of fluorescent-labeled microorganisms raised against microgram amounts of mucins spotted on nitrocellulose was developed. The adhesion of various probiotic, commensal and pathogenic microorganisms was evaluated on a panel of human purified gastrointestinal mucins and compared with that of commercially available pig gastric mucins (PGM) and of mucins secreted by the colonic cancer cell line HT29-MTX. The latter two proved to be very poor indicators of adhesion capacity on intestinal mucins. Our results show that the nature of the sialylated cores of *O*-glycans, determined by MALDI MS-MS analysis, potentially enables sialic acid residues to modulate the adhesion of microorganisms either positively or negatively. Other identified factors affecting the adhesion propensity were *O*-glycan core types and the presence of blood group motifs. This test should help to select probiotics with enhanced adhesion capabilities as well as deciphering the role of specific mucin glycotopes on microbial adhesion.

## 1. Introduction

According to the FAO/WHO (Food and Agriculture Organisation of the United Nations/World Health Organisation), probiotics are live microorganisms which, when taken in adequate amounts, can provide health benefits to the host [1]. Most current probiotics used belong to the genera *Lactobacillus* and *Bifidobacterium*, with a few other species, such as *Bacillus* sp., *Escherichia coli*, and *Streptococcus* sp. Probiotics are known to exert numerous beneficial effects, helping to prevent or treat a variety of health problems or diseases, such as diarrhea caused by infections or antibiotics, irritable bowel syndrome, inflammatory bowel disease and allergic disorders. They play a role in reducing the incidence of common upper respiratory tract infections and help to manage vaginal infections. They are presumed to modulate the commensal intestinal flora. The mechanisms of action displayed by these phylogenetically diverse microorganisms range from immunomodulation [2,3] and epithelial barrier maintenance [4] to direct antagonism with pathogens via bacteriocin production [5,6] and competitive exclusion [7,8]. Short chain fatty acids, such as formic, acetic, propionic, butyric and lactic acids, are produced by probiotics during carbohydrate catabolism and play an important role in the decrease of pH that inhibits the growth of enteric pathogens [9,10].

As for the intestinal commensal microbiota residing in close association to the host epithelial mucus, adhesion to intestinal mucosa is expected to be a crucial property for ingested probiotics. The increased host–bacterial interactions favoured by bacterial adhesion are thought to enable health benefits to the host. Probiotics can also prevent the attachment of pathogens to the intestinal mucus [11].

The mammalian gastrointestinal tract is covered by mucus, a viscoelastic gel that lines and protects the intestinal epithelium, separating it from the lumen content. The large intestine is covered with a bilayer of mucus, with the outer layer providing a habitat for bacteria, whereas the inner layer maintains them at a safe distance from the epithelial surface and is devoid of microorganisms under healthy conditions [12]. The mucus thickness varies along the intestine, being thicker in the colon than in the small intestine [13]. This mucus traps and transports bacteria and is a rich source of nutrients used for bacterial metabolism and growth [14]. The main constituents of mucus are mucins, which are produced, stored and released by goblet cells. Mucins are large glycoproteins in which the glycans constitute more than 80% of the molecular mass. To date, 20 human MUC genes have been assigned to the MUC gene family, some of them belonging to the secreted gel-forming mucin family, whereas the others are classified in the membrane-bound family [15].

The expression of mucins is organ- and tissue-dependent. In the adult human stomach, two mucins, MUC5AC and MUC6, are secreted by different categories of cell types. MUC5AC is highly expressed in mucous neck cells at the surface of the gastric epithelium [16,17], whereas MUC6 is expressed in mucous neck cells and the principal cells of the body and in pyloric glands of the antrum [16,18,19]. In the small intestine and the colon, the mucus layer mainly consists of MUC2 [12], even though low levels of MUC5B can also be found [20].

In humans, MUC2 is coated with more than 100 different *O*-linked glycan chains [21,22,23]. Mucin oligosaccharides can serve both as binding sites and energy sources for intestinal microbiota and the differences in mucin glycosylation determined along the intestine [22] and between individuals can influence the tropism of some bacteria for specific regions of the gastrointestinal tract [24] as well as the host specificity in terms of microbiota [25].

*O*-glycosylation of mucins is initiated by the addition of *N*-acetyl-galactosamine (GalNAc) to Ser and Thr residues and further proceeds with sequential addition of monosaccharides: galactose (Gal), *N*-acetyl-glucosamine (GlcNAc), Fucose (Fuc), GalNAc and sialic acid residues [26]. Sulphate residues are also found in the periphery of *O*-glycans. Three regions may be distinguished within mucin *O*-glycans, in direct correlation with their biosynthesis: the core, backbone and peripheral regions [27]. The core region corresponds to the first GalNAc mono- or di-substituted with α- or β-Gal, β-GlcNAc and α-GalNAc. This complex pattern of core types can be further complicated by the addition of *N*-acetyl-neuraminic acid (NeuAc) which is potentially linked to the first GalNAc residue. In animals, *N*-acetyl-glycolyl acid residues can be found in place of NeuAc. The backbone regions consist of alternating Gal and GlcNAc in β1–3 (for the type 1 chains) and β1–4 (for the type 2 chains) linkages [28]. Fuc, Gal, GalNAc and NeuAc residues are the four monosaccharides found at the periphery or at internal positions of the polylactosamine backbones. Sulphate residues are also found to substitute Gal, GalNAc or GlcNAc residues. The peripheral region characterizes the mucin by conferring a specific charge to the molecule as well as having antigenic properties.

Glycosylation of mucins varies along the gastrointestinal tract of healthy human individuals. In the human stomach, more than 70 different oligosaccharides are carried by mucins. These are mostly neutral and highly fucosylated based on a core of 2–Galβ1–3[GlcNAcβ1–6]GalNAc [29].

In contrast to gastric mucins, human intestinal mucin *O*-glycans are mainly based on sialylated core 3 structures (GlcNAcβ1-3GalNAc) [22]. Extensive differences in the glycosylation patterns of mucins along the intestinal tract have been described [30,31], characterized by the presence of decreasing gradients of fucose and ABH blood group expression from the ileum to the rectum as well as an increasing acidic gradient [21]. The high degree of diversity in the expression of glycans in the different parts of the intestine creates an enormous repertoire of potential binding sites for microorganisms that could explain the regio-specific colonization of bacteria in the human gut.

In this context, we developed a rapid and sensitive test for the characterization of the binding capacities of probiotic microorganisms to human purified mucins spotted on a nitrocellulose membrane, allowing us to compare the adhesion of probiotics to mucins with that of commensal bacteria known to colonize mucus and pathogenic bacteria known to interact with the epithelial mucosa and/or mucins. Five different probiotic bacteria and one yeast were used in this study. The *E. coli* strain, Nissle 1917, has proven to be efficient in the treatment for maintaining remission in ulcerative colitis [32] and helps to stop acute diarrhea in infants and toddlers [33]. Lactobacilli have long been considered to be good for health and due to their beneficial and protechnological properties, numerous *Lactobacillus* species are used as probiotics with recognized effects in the treatment of gastrointestinal disease [34]. Members of the *Lactobacillus plantarum* species have been found to reduce the concentration of cholesterol and fibrinogen and reduce the risk of cardiovascular disease and atherosclerosis [35]. *Lactobacillus rhamnosus* displays a wide array of probiotic properties, including the reduction of diarrhea, atopic eczema and respiratory infections [36]. *Lactobacillus paracasei* improve nutrition and aid gastrointestinal and respiratory disease prevention and therapy [37]. The *Lactobacillus casei* strain, Shirota, may favourably affect metabolic abnormalities in obese subjects [38]. The yeast, *Saccharomyces cerevisiae* CNCM I-3856, is recommended to improve the symptoms of irritable bowel disease [39].

The *E. coli* strain, K12, was used as a positive control for adhesion to mucins, as it is a commensal bacterium that colonizes the intestinal mucosa. Three intestinal pathogens were evaluated, all of them expressing outer membrane proteins able to bind to mucosa: *Yersinia enterocolitica*, which causes abdominal pain, diarrhea, vomiting and weight loss [40,41]; *Shigella sonnei*, which is responsible for bloody diarrhea [42] and *Salmonella enterica*, which is among the most important agents responsible for food outbreaks occurring worldwide [43].

The correlation between the adhesion levels of each microorganism tested and the *O*-glycan pattern of mucins points to important features that mediate the specific binding action of each studied species.

## 2. Methods

### 2.1. Bacteria and Yeast Culture

Lactobacillus rhamnosus, Lactobacillus paracasei and Lactobacillus plantarum were grown in Lactobacilli MRS broth (Sigma-Aldrich, St Louis, MO, USA). E. coli K12 and Nissle 1917, Salmonella enterica, Shigella sonnei and Yersinia enterocolitica were grown in LB medium. All of the abovementioned strains were a kind gift from the “Institut de microbiologie du CHRU de Lille”. Saccharomyces ceraevisiae (sold by Lesaffre, Marcq-en-Barœul, France, under the name Ibsium) was grown in YEP medium (Yeast Extract Peptone: peptone 20 g/L, yeast extract 10 g/L) complemented by 2% glucose. All cultures were performed for 16 h at 37 °C under shaking. L. casei Shirota was directly obtained by centrifugation of commercially available Yakult at 3500 g for 10 min, followed by two washes with PBS. This product claims to contain exclusively L. casei Shirota.

### 2.2. Isolation and Purification of Mucins from Human Tissues and Cell Lines

Human mucins were purified from different regions of the gastrointestinal tract and from malignant ovarian cysts. Human adult stomachs, jejunums, ileums and colons were obtained from the France Transplant Association from kidney donors, in accordance with protocols approved by the National Ethical Committee. Samples of mucosa were snap-frozen in liquid nitrogen and stored in liquid nitrogen until use. Malignant ovarian cyst mucins were collected by Dr Bara in accordance with protocols approved by the National Ethical Committee. The use of human tissues for this study was approved by the local hospital ethics committee and the French Ministry of Higher Education and Research (DC-2008-242).

Mucins from HT29-MTX were obtained by collecting the culture medium of cells after 21 days of growth. HT29-MTX were maintained in standard Dulbecco’s modified Eagle’s minimal essential medium supplemented with 10% (*v*/*v*) heat-in-activated foetal calf serum, 2 mM L-glutamine, 100 unit/mL penicillin and 100 unit/mL streptomycin at 37 °C in 5% CO_2_.

Mucins were solubilized in 4 M guanidine chloride solution containing 5 mM ethylenediaminetetraacetic acid, 10 mM benzamidine, 5 mM *N*-ethylmaleimide, 0.1 mg/mL soy bean trypsin inhibitor and 1 mM phenylmethanesulfonyl fluoride.

CsCl was added to an initial density of 1.4 g/mL and mucins were purified by isopycnic density-gradient centrifugation (Beckman Coulter LE80 K ultracentrifuge; 70.1 Ti rotor, 417,600 g at 15 °C for 72 h). Fractions of 1 mL were collected from the bottom of the tube and analyzed for periodic acid-Schiff (PAS) reactivity and density. The mucin-containing fractions were pooled, dialyzed against water and lyophilized. Pig gastric mucin type III (PGM) was supplied by Sigma-Aldrich.

### 2.3. Release of Oligosaccharides from Mucin by Alkaline Borohydride Treatment

Mucins were submitted to β-elimination under reductive conditions (0.1 M KOH, 1 M KBH4 for 24 h at 45 °C) and the mixture of oligosaccharide alditols was dried on a rotavapor (Buchi, Flawil, Swisserland) at 45 °C. Borate salts were eliminated by several co-evaporations with methanol before purification by cation exchange chromatography (Dowex 50 × 2, 200–400 mesh, H + form).

### 2.4. Permethylation and Mucin Glycosylation Analysis by MALDI TOF MS

Permethylation of the mixture of oligosaccharide alditols was carried out with the sodium hydroxide procedure described by Ciucanu and Kerek [44]. Briefly, oligosaccharides were incubated for 2 h at room temperature in 200 µL of dimethylsulfoxide, a spatula tip of sodium hydroxide and 300 µL of iodomethane. After derivatization, the reaction products were dissolved in 1 mL of acetic acid solution (5%, *v*/*v*) and further purified on a C18 Sep-Pak column (Oasis HLB, Waters, Milford, MA, USA). The cartridge was preconditioned with 3 mL of methanol. After washing the cartridge with 4 mL of 5% methanol, glycans were eluted in 4 mL of methanol. Permethylated oligosaccharides were analyzed by MALDI TOF MS in positive ion reflective mode as [M+Na]^+^. Quantification through the relative percentage of each oligosaccharide was calculated based on the integration of peaks on MS spectra.

### 2.5. 1-D Bacterial Overlay

The 1-D bacterial overlay procedure was adapted from Odenbreit et al. [45]. In brief, purified mucins (1μg/μL in PBS or 4 M guanidine chloride in PBS) were spotted on dry nitrocellulose membranes using a Bio-Dot SF (Biorad Hercules, CA, USA) which were saturated with PFBB (Protein Free Blocking Buffer) (Thermo-Scientific, Waltham, MA, USA) for 1 h. Bovine serumalbumin at 1 µg/µL was used as a negative control. Bacteria were labeled either with 2.5 µg/mL DAPI or 50 µM syto9 in PBS for 15 min or with FITC at 0.1 mg/mL (for Gram− bacteria) or 0.2 mg/mL (Gram+ bacteria) in carbonate buffer (NaCl 0.15 M, NaHCO_3_ 0.1 M) for 1 h. Labeled bacteria were collected by centrifugation at 3000× *g* for 5 min, washed three times in PBS, suspended in 1 mL of blocking buffer and added to the membrane in blocking buffer. In the case of FITC, an additional step, involving 2 h of incubation in blocking solution before the final three washes, was included to reduce the background signal. After incubation for 1 h at room temperature in the dark, followed by three washes of membranes in PBS containing 0.5% Tween 20, the fluorescence of adherent bacteria was detected by a ChemiGenius 2 imaging system (Syngene, Frederick, MD, USA). Mucins were chemically desialylated for 1 h at 80 °C in a 0.05 M trifluoroacetic acid (TFA) solution.

### 2.6. Statistical Analysis

Data are reported as means ± SDs of at least 3 replicates. Student’s *t*-test was used for statistical analysis.

## 3. Results

### 3.1. Development of a New Assay to Evaluate the Adhesion of Microorganisms on Mucins

**Figure 1:**
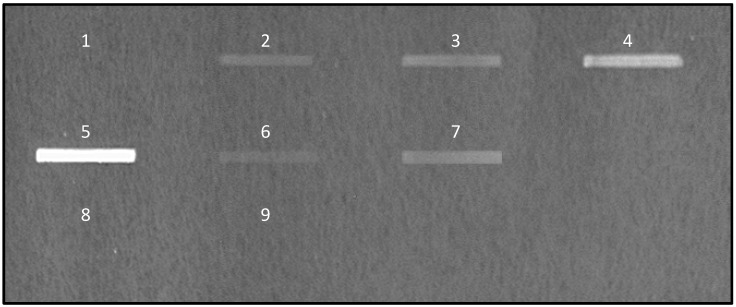
Adhesion of DAPI-labeled *L. paracasei* on mucins. Purified mucins were spotted on nitrocellulose membrane before incubation with DAPI-labeled bacteria. Fluorescence signaling was detected using the Chemigenius2 Bio-imaging system before quantification with GeneTools software. Spotted samples were BSA (1); pig gastric mucin (2); and human mucins purified from ileum (3); jejunum (4); colon (5); stomach (7) and ovarian cysts (8). After chemical removal of sialic acids, mucins from human colon and ovarian cysts were spotted respectively at positions (6) and (9).

Teams working on microorganism adhesion to mucins mainly use 96-welled plates coated with mucins. These assays usually require high amounts of mucins and are not suitable for bacteria like *Pseudomonas aeruginosa* which have a tendency to adhere to plastic. To circumvent these limitations, a new assay was developed using nitrocellulose membrane as a support. Mucins were spotted on a nitrocellulose membrane that was further blocked with a saturating agent to avoid the unspecific binding of microorganisms. Several blocking reagents were tested in this study—5% milk powder in PBS, 0.5% BSA in PBS and PFBB (Pierce protein free blocking buffer) with which the best signal/noise ratio was obtained. Bacteria were labelled with fluorescent dyes before incubation on the membrane. DAPI was preferred to FITC and syto9, which, respectively need long labelling times and are associated with fast loss of the fluorescent, as already mentioned by Stiefel et al. [46]. DAPI was therefore chosen for further experiments. The results obtained with *L. paracasei* are shown in Figure 1. Clear differences in fluorescence can be observed depending on the mucin coated, with mucins from the colon indicating much higher adhesion than mucins from other sources. No signal was detected with the negative control, bovine serum albumin (BSA).

**Figure 2:**
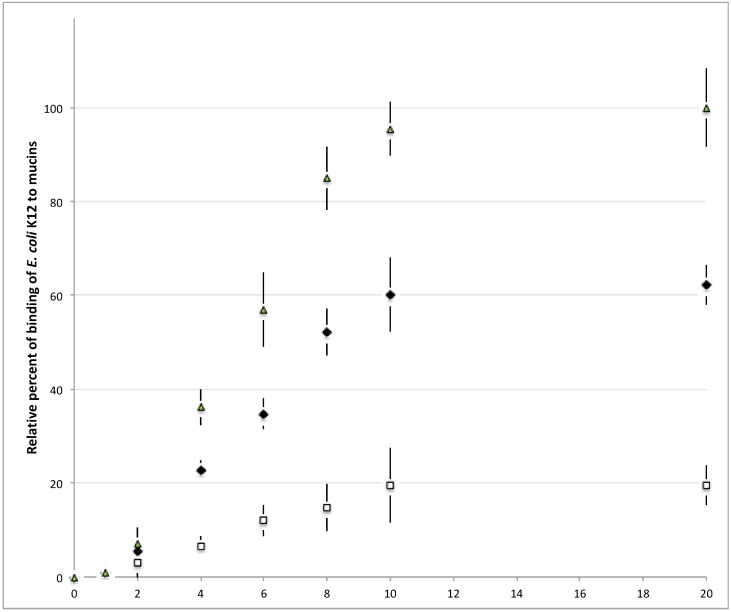
Relationship between the intensity of fluorescence and the number of mucins spotted. Mucins from the human colon (∆), jejunum (◆) and ileum (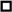) were spotted at different quantities on nitrocellulose membrane and the signal of bound fluorescent-labeled *E. coli* K12 was quantified. Above 20 μg of mucins per spot of 9 mm^2^, the signal reaches a plateau.

To establish the optimal conditions required to obtain a strong signal without using excessive amounts of purified mucins, mucins from human jejunums, ileums and colons were spotted at quantities ranging from 1 μg to 20 μg and the signal obtained with DAPI labelled *E. coli* K12 was quantified. As shown in Figure 2, above 2 μg of coated mucins, the signal proportionally increased with the amount of mucin spotted on the membrane until approximately 10 μg of mucins. The level of fluorescence only slightly increased between 10 μg and 20 μg, suggesting that it is not necessary to increase the amount of mucin spotted on the membrane above this range, as the gain of signal will then be negligible. For the following experiments, mucins were systematically spotted at a quantity of 20 μg.

### 3.2. Adhesion of Microorganisms on Commercially Available Pig Gastric Mucins (PGM) or Mucins from HT29-MTX Cell Lines Does Not Reflect Adhesion on Human Intestinal Mucins

Most assays developed to evaluate the capacity of adhesion of bacteria or yeasts on mucins use PGM as the standard material, taking for granted that the adhesion on this material will reflect the overall affinity of the microorganisms for mucins. As seen in Figure 1, the results obtained with PGM are poorly informative. Even though a signal is detectable with PGM for all tested bacteria while no signal is seen with BSA used as a negative control, the difference of binding between bacteria described as only transiently passing through the gastrointestinal tract (*L. casei Shirota*) and bacteria known to colonize mucus (the commensal *E. coli K12*) is very low.

**Figure 3:**
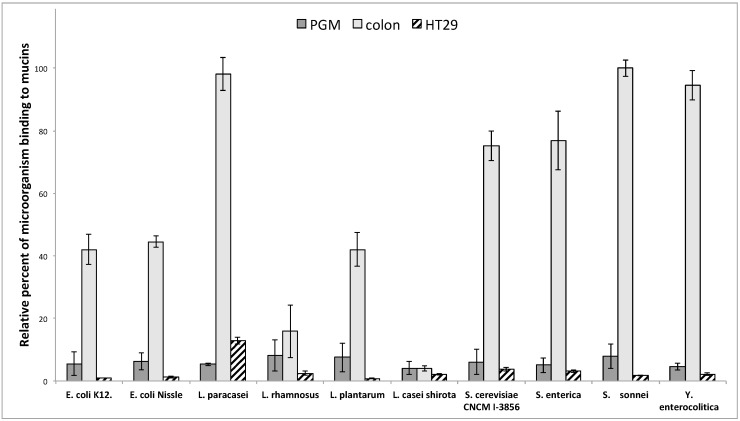
The adhesion of various probiotic and pathogenic microorganisms on commercially available pig gastric mucins or mucins from HT29-MTX cell lines does not reflect the adhesion on human intestinal mucins. The binding of DAPI-labeled microorganisms to pig gastric mucins (dark grey), human purified colonic mucins (light grey) and mucins from HT29-MTX (striped) was quantified by slot-blot overlay assays. No correlation was observed between the level of adhesion of microorganisms to colonic mucins and to HT29-MTX or PGM. Data shown is a representative experiment ± SD of three replicates.

Mucus and mucins from the mucus-secreting HT29-MTX intestinal epithelial cell lines are also widely used to investigate the adhesion of microorganisms. In this study, we first compared the binding of bacteria or yeasts on PGM, HT29-MTX and purified human intestinal mucins. As seen in Figure 3, the level of adhesion obtained with human intestinal mucins was considerably higher than with PGM or HT29-MTX. Given the reported differences in binding, which vary depending on the origin and site of the mucin utilized for binding assays, care should be taken in the interpretation of the data when using PGM or HT29-MTX as a unique source of mucin to assay bacteria binding to intestinal mucin. For example, no real difference in the level of adhesion with PGM or HT29-MTX is detected between *L. casei* shirota and *Y. enterocolitica* when the latest clearly binds to intestinal mucins contrary to the first.

As shown in Figure 3, the microorganisms used in this study displayed different patterns of binding to human intestinal mucins. The three pathogens, *S. enterica*, *S. sonnei* and *Y. enterocolitica*, presented a high capacity for adhesion to colonic mucins. The two strains of *E. coli*, the commensal *E. coli* K12 and the probiotic *E. coli* Nissle 1917, also showed a high rate of binding to human intestinal mucins. Among the other probiotic bacteria tested, only *L. casei* Shirota exhibited no significant adhesion to intestinal mucins. *L. rhamnosus* showed moderate binding, whereas all other bacteria and yeast (*S. cerevisiae*) displayed strong binding capacities.

**Figure 4:**
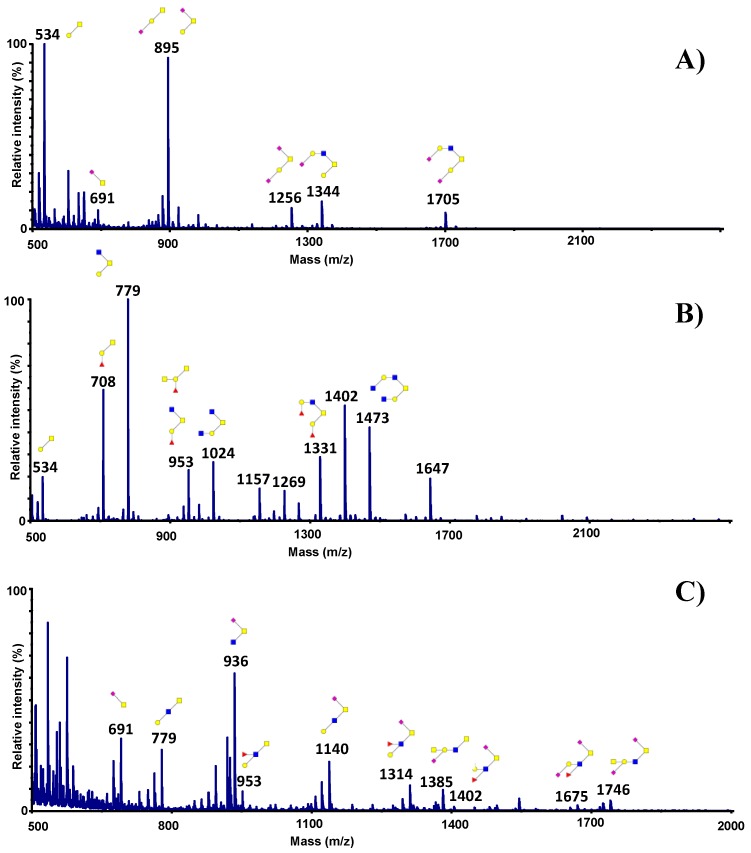
MS spectra of permethylated *O*-glycans isolated from mucins purified from HT29-MTX (**A**); from pig gastric mucins (**B**) and from human colonic mucins (**C**). Mucin *O*-glycans were released from the protein backbone and permethylated before analysis by MALDI-TOF mass spectrometry in the positive ion mode [M+Na]^+^. Monosaccharide symbols are used according to the Consortium for Functional Glycomics (CFG) nomenclature. Key: fucose (red triangle), GlcNAc (blue square), sialic acid (purple diamond), galactose (yellow circle), GalNAc-ol (yellow square) and sulfate residue (S). Note that for simplified comprehension, only the structure corresponding to the major isomer was drawn on the MS spectrum of PGM. Other isomers with the same monosaccharide composition might be present.

Bacterial adhesion to mucins mostly mediated by interaction between mucin *O*-glycans and bacterial adhesins. Because bacteria showed differences in their patterns of binding to mucins, we next compared the repertoire of glycosylation of PGM, HT29-MTX and human intestinal mucins. Oligosaccharides were released by reductive β-elimination from the protein backbone, permethylated and analysed by MALDI-TOF-TOF mass spectrometry in the positive ion mode (Figure 4). HT29-MTX mucin *O*-glycans are mainly composed of Thomsen-Friedenreich (TF) (Galβ1-3GalNAc) at *m*/*z* 534 and sialyl TF antigens (NeuAcα2-3Galβ1-3GalNAc or Galβ1-3(NeuAcα2-6)GalNAc) at *m*/*z* 895, each representing around 35% of the whole *O*-glycans detected in these cells. Fifty-five percent of oligosaccharides were sialylated, with major sialylated glycans identified at *m*/*z* 691, 895, 1256, 1344 and 1705. The ions at *m*/*z* 691 corresponded to sialyl Tn antigens (NeuAcα2-6GalNAc) and the ions at *m*/*z* 1256 were disialylated TF antigens (NeuAcα2-3Galβ1-3(NeuAcα2-6)GalNAc). The two other major sialylated glycans corresponded to ions at *m*/*z* 1344 and 1705 on the MS spectrum (Figure 4A). They were both based on a core 2 glycan (Galß1-3(GlcNAcβ1-6)GalNAc) elongated with a galactose residue on the GlcNAc in the upper branch and carrying one (for the ion at *m*/*z* 1344) or two sialic acid residues (for the ion at *m*/*z* 1705). Around 75% of all identified glycans were based on a core 1 structure (Galβ1-3GalNAc) further elongated by GlcNAc residues or substituted with fucose residues. 

Pig gastric mucin oligosaccharides have been well characterized in previous studies [47,48,49]. They contain a high proportion of core 1 and core 2 *O*-glycans, as illustrated on the MS spectrum by the ions at *m*/*z* 534 and 708 for the core 1 glycans and the ions at *m*/*z* 1024, 1331, 1473 and 1647 for the core 2 glycans. Many ions correspond to several structural isomers based either on core 1 or core 2, as shown for the ions at *m*/*z* 779, 953, 1157 and 1402. Most of the oligosaccharides from pig gastric mucins are elongated by type 2 lacNAc chains, capped or not, by Fucα2 linked to Gal to form blood group H antigen, as seen in the Figure 4B for the ions at *m*/*z* 708, 1157 and 1331. An additional GalNAc may be α1-3 linked to the galactose residue to give a blood group A antigen. This was the case for the ions at *m*/*z* 1402 and 1647, for example. Apart from sialyl TF antigens, which are expressed at a very low level, no sialylated oligosaccharides were recovered in pig gastric mucins.

On the contrary, human intestinal mucins are highly acidic and almost all the glycans are based on a core 3 structure (GlcNAcβ1-3GalNAc), as we have previously published [21,22]. Moreover, many of them had sialic acid α2-6 residues linked to the first GalNAc, as illustrated on the MS spectrum by the ions at *m*/*z* 936, 1140, 1314, 1675 and 1746 (Figure 4C). Only few minor glycans possessed core 1 or core 2 structures. Numerous intestinal mucin *O*-glycans carry blood group and Lewis antigens, as well as more specific antigens like Sda/Cad determinants (GalNAcβ1-4(NeuAcα2-3)Galβ1-), for example, the ion at *m*/*z* 1385. 

### 3.3. Influence of Sialic Acid Residues and/or Core Structure on Bacterial and Yeast Adhesion to Human Mucins

**Figure 5:**
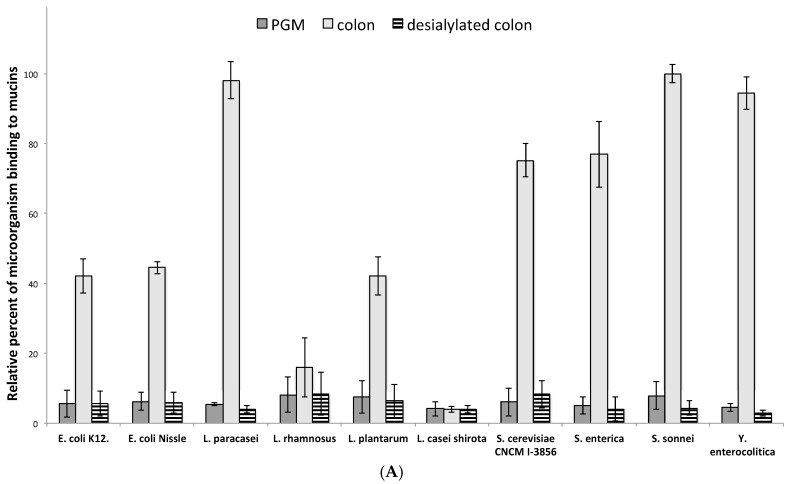

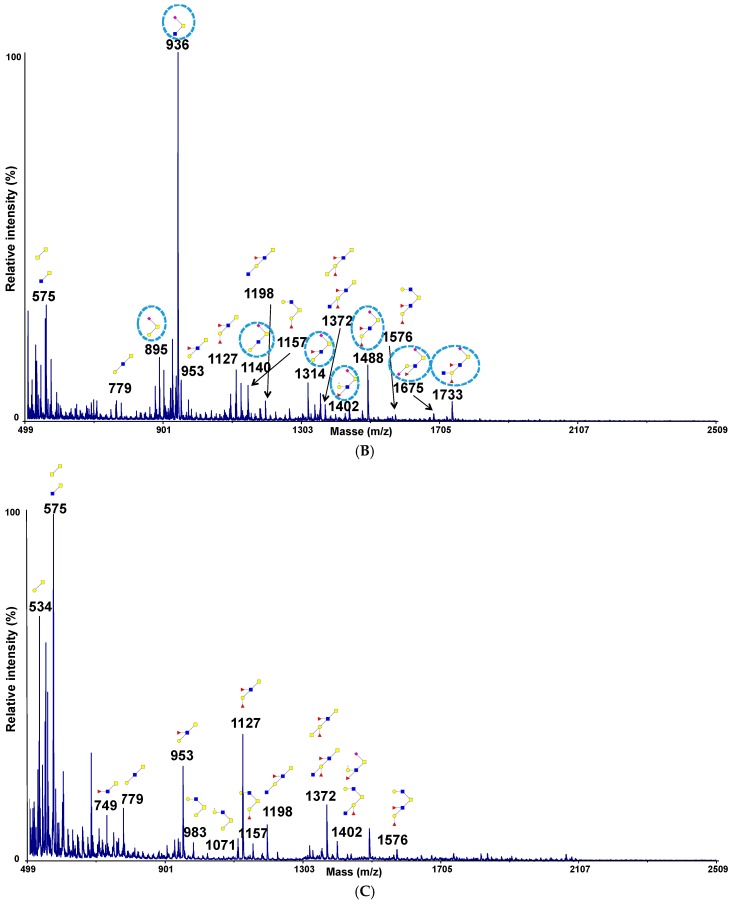
Influence of sialic acid residues on the adhesion of microorganisms to human mucins. (**A**) Bacteria and yeast preferentially bind to native human colonic mucins. The binding of DAPI-labeled microorganisms to pig gastric mucin (dark grey), human purified colonic mucins (light grey) and their desialylated counterparts (striped) was quantified by slot-blot overlay assays. A strong decrease of binding was observed for all microorganisms tested after chemical desialylation of mucins, the level of adhesion reaching that of PGM. Data shown is a representative experiment ± SD of three replicates. (**B**,**C**) MS spectra of permethylated *O*-glycans isolated from native (**B**) and desialylated human colonic mucins (**C**), acquired in the positive ion mode [M+Na]^+^. Monosaccharide symbols are used according to the Consortium for Functional Glycomics (CFG) nomenclature. Key: fucose (red triangle), GlcNAc (blue square), sialic acid (purple diamond), galactose (yellow circle), GalNAc-ol (yellow square) and sulfate residue (S).

To determine if sialylation could be a key factor in the binding of microorganisms to mucins, we next chemically desialylated human intestinal mucins and compared the level of adhesion of bacteria and yeast to native mucins and their desialylated counterparts (Figure 5A). The removal of sialic acids resulteds in a dramatic decrease in the level of adhesion, ranging from 47% for *L. rhamnosus* and 89% for the tested strain of *S. cerevisiae* to up to 96% for *L. paracasei* and 97% for *Y. enterocolitica*. For all the microorganisms tested, the level of binding to desialylated human intestinal mucins was near the same as that of native PGM.

**Figure 6:**
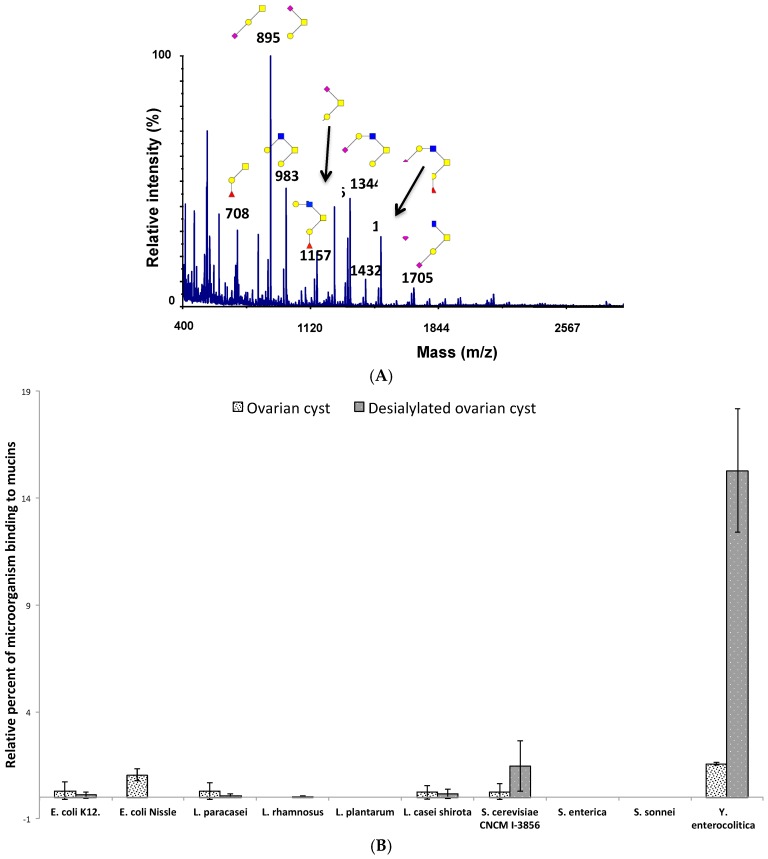
Not all types of sialylated mucin *O*-glycans are ligands for microorganisms. (**A**) MS spectrum of permethylated *O*-glycans isolated from human malignant ovarian cyst mucins, acquired in the positive ion mode [M+Na]^+^. Monosaccharide symbols are used according to the Consortium for Functional Glycomics (CFG) nomenclature. Key: fucose (red triangle), GlcNAc (blue square), sialic acid (purple diamond), galactose (yellow circle), GalNAc-ol (yellow square) and sulfate residue (S). (**B**) Binding of DAPI-labeled microorganisms to human purified native ovarian cyst mucins (white, black dots) and their desialylated counterparts (dark grey, white dots) was quantified by slot-blot overlay assays. Sialic acid residues from ovarian cyst mucin *O*-glycans were not recognized by microorganisms. Chemical desialylation of ovarian cyst mucins significantly increased the binding of *Y. enterocolitica*. Data shown is a representative experiment ± SD of three replicates.

In human intestinal mucins, sialic acids are mainly α2-6 linked to the first GalNAc, which is also substituted by a GlcNAc residue β1,3 linked to form a core 3 glycan. To determine whether only the sialic acid residues are important for the binding of microorganisms or if the core structure can play a role in the recognition, we next evaluated the binding of bacteria to malignant human ovarian cyst mucins. As shown in Figure 6A, ovarian cyst mucin *O*-glycans exhibited a high proportion of sialylated glycans (more than 50% of all the *O*-glycans identified). However, in contrast to intestinal mucin oligosaccharides, sialylated glycans from ovarian cysts were mainly based on a core 1 structure, with a small proportion of sialylated core 2 glycans. As depicted by Figure 6B, most tested microorganisms showed no significant adhesion with the *O*-glycan structures found on native mucins from ovarian cysts. Only a low level of adhesion on these mucins was detected for *E. coli* Nissle 1917 and *Y. enterocolitica*. When treated under mild acidic conditions to remove sialic acids from *O*-glycans, ovarian cystic mucins gained the capacity to be used as a ligand by two of the tested microorganisms, the bacteria *Y. enterocolitica* and to a lower extent, the yeast *S. cerevisiae* CNCM I-3856. These results seem to indicate that depending on the type of core harboured by mucin *O*-glycans, sialic acid residues can either constitute a crucial part of the binding site or mask a potential site of adhesion.

### 3.4. Adhesion of Microorganisms to Mucins Purified along the Gastrointestinal Tract

**Figure 7:**
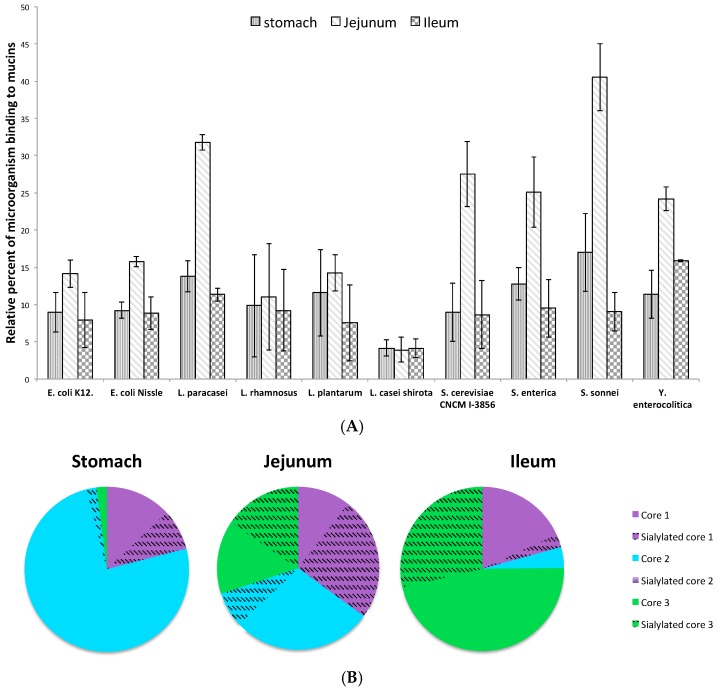
Adhesion of microorganisms along the gastrointestinal tract. (**A**) Binding of DAPI-labeled microorganisms to human purified mucins from the stomach (vertical strips), jejunum (oblique strips) and ileum (chequerwise) was quantified by slot-blot overlay assays. Microorganisms bind preferentially to colonic mucins and to jejunal mucins. Data shown is a representative experiment +± SD of three replicates. (**B**) Structural features of human gastric, jejunal and ileal mucin glycosylation. Schematic illustration of the repartition of sialylated and non-sialylated oligosaccharides based on core 1, 2 or 3 glycans, carried by mucins.

We next evaluated the binding of microorganisms to human mucins purified all along the gastrointestinal tract, i.e., human stomach, human jejunum and ileum (small intestine) and human colon. As shown in Figure 7A, the strongest binding was always observed for human colonic mucins, whatever the microorganism tested. We noticed a 1.4 to 4.5-fold increased adhesion level to colonic mucins compared to human jejunal mucins, the difference of adhesion being the lowest for *L. rhamnosus* and the highest for *Y. enterocolitica*. Each microorganism tested depicted different patterns of binding to human mucins. For example, the pathogenic bacteria *S. sonnei* showed very strong binding to colonic mucins (compared to the other bacteria tested), a 2.5-fold decreased adhesion to jejunal mucins, a 5.8-fold decreased adhesion to gastric mucins and an 11-fold decrease for ileal mucins. On the contrary, the adhesion of *L. rhamnosus* to human gastrointestinal mucins was very weak and little difference was observed between mucins.

Compared to human colonic mucins, the binding of microorganisms to gastric and ileal mucins remains weak, with around a 5 to 11-fold decrease for all tested bacteria and yeast.

To better understand the difference in the binding of microorganisms to human jejunal mucins compared to ileal and gastric mucins, we characterized the glycosylation of the different mucins (Figure 7B). Human gastric mucins were mainly based on core 2 glycans. Most of the oligosaccharides were neutral and highly fucosylated. Only 10% of the structures were acidic, carrying sialic acid residues. Among these sialylated oligosaccharides, sialyl TF and disialyl TF antigens (NeuAcα2-3Galβ1-3(NeuAcα2-6)GalNAc), based on a core 1 structure, were the most expressed. Around 3% of oligosaccharides were short sialylated core 2 glycans. A major feature of human gastric mucins was the expression of blood group antigens, carried by around 80% of the *O*-glycans. In the jejunum, glycans were either based on a core 1, core 2 or core 3 structure. Around 45–50% of *O*-glycans were sialylated, with mainly core 1 and core 3 sialylated oligosaccharides linked at α2-3 to Gal residues or at α2-6 to the first GalNAc. Around 35–40% of oligosaccharides carried blood group antigens. In the ileum, oligosaccharides were predominantly neutral and highly fucosylated, but, in contrast to gastric mucins, they were mainly based on a core 3 structure, with some glycans based on a core 4 structure (GlcNAcβ1-3(GlclNAcβ1-6)GalNAc). Around 55% of the oligosaccharides carried blood group and Lewis determinants and only 30% of glycans were sialylated. Most of the sialic acid residues were α2-6 linked to the first GalNAc and carried by a core 3 structure.

## 4. Discussion

The large intestine is lined by two layers of mucus, the innermost of these remaining devoid of microorganisms under healthy conditions. This means that intestinal cells are usually not directly exposed to probiotic or commensal intestinal flora. For this reason, evaluating the efficacy of probiotics on protecting the human intestinal mucosa requires the use of either biological samples or the disposal of cell culture models covered by mucus, which is not the case for the Caco2 cells very often used in studies aimed at evaluating the adhesion of probiotics to the gastrointestinal tract.

Other studies regarding the adhesion properties of potential probiotics within the mucus have either been directly performed on HT29 cell cultures or performed on mucins purified from this material. Our results show that no interpolation can be made regarding the adhesion on colonic mucins from the binding obtained on HT29 purified mucins. HT29 cells are derived from a colic tumour, and it has been shown that glycosylation is strongly affected by carcinogenesis. Indeed, HT29 secreted mucins mostly harbour TF, sialyl TF and disialyl TF antigens based on a core 1 structure, whereas the predominating healthy colonic structures are based on core 3 glycans. Moreover, mucins from mucus secreting HT29 cells are mainly MUC5AC mucins whereas major secreted mucins from the intestine are MUC2 mucins.

An alternative to HT29 mucins commonly found in the literature is pig gastric mucin (PGM). This mucin, also called PGM type-II or type-III, is often erroneously taken as MUC2. Here, again, the results presented show that even if this commercially available mucin is very convenient to use, the adhesion levels obtained with this material do not allow conclusions to be drawn on the binding propensity of microorganisms for intestinal mucins.

The bacterial overlay presented here is suitable for the performance if a fast screen, allowing the identification of probiotics with high mucus adhesion propensity. Nevertheless, it does not have the ability to proclaim that a given microorganism will be able to bind in vivo to the areas of the gastrointestinal tract from which purified mucins have been extracted. Indeed, the main criteria for bacteria to be able to reside in a given area of the intestine, is that they are able to reach the mucus of this organ as a living organism. To exert health benefits, the minimum concentration of live probiotic bacteria at point of delivery should be above 10^7^ cfu mL^−1^ [50]. The viability of probiotics must therefore not be altered by the passage through the adverse acidic environments of the stomach and the entry into the duodenum that not only involves a change in pH, but also exposes microorganisms to bile salts which act as detergents, causing cell damage and cytotoxicity [51,52]. Actually, less than 10% of the strains tested can grow in the gastrointestinal tract [53] and gut microorganisms have evolved highly conserved mechanisms for tolerance to gastrointestinal stresses [54,55]. In some cases, the adhesive properties of bacteria to mucins are positively affected by the passage through the stomach and duodenum and their adhesion propensity may be increased compared to the same types of bacteria that are not submitted to the harsh pH treatments [56].

The dramatic loss of microorganism adhesion observed when intestinal mucins were chemically desialylated (Figure 5A) emphasizes the role played by sialic acids in this process. The involvement of sialic acid in the binding of gut microorganisms to mucins is well documented. For instance, the recently described adhesin domain, CBM40, present in the human gut symbionte *Ruminococcus gnavus* is specific towards sialoglycans with a millimolar binding affinity towards α2,3- or α2,6-sialyllactose. It also mediates adhesion to mucins [57]. In another study [58], the crucial role played by sialic acid residues bound to mucin *O*-glycans on the adhesion observed for two lactobacilli strains and one bifidobacteria strain was assessed. Lactobacillus exhibits numerous mechanisms for adhesion to mucus, among which mucus-binding proteins (MUBs) have been well characterized. The MUB proteins contain repeated functional domains (Mub repeats), which share homology with the Pfam-MucBP (mucin-binding protein) domains (PF06458). Mub repeats have been shown to adhere to pig gastric mucins and hen intestinal mucus [59]. Adhesion assays of MUB from *Lactobacillus reuteri* on mammalian tissue sections and a mucus-secreting intestinal cell line demonstrated the binding of MUB to sialylated mucin glycans [60]. The importance of sialic acid in mucin binding has also been assessed for bacteroidetes, whose adhesin NanU has been demonstrated to bind to Neu5Ac with high affinity in *Bacteroides fragilis* [61].

The importance of sialic acids in adhesion to mucins also applies to pathogenic bacteria. The best-documented example is *Helicobacter pylori* which shows the influence of blood group motives containing *O*-glycans at the surface of the gastric mucosa, recognized by the adhesin BabA. Upon infection by *H. pylori*, the rate of sialylated mucins, scarce in a healthy stomach, increases significantly [62,63,64]. Glycans harboured on these mucins present the motif, SLex, the specific ligand for sialic acid-binding adhesin (SabA) [65]. Pathogens like *Streptococcus gordonii*, *Streptococcus sanguinis* and *Streptococcus mitis* present the capacity to adhere to sialylated mucins via serine rich region proteins (SRR proteins) [66] or Hsa adhesin [67]. Our results show that the nature of the sialylated core of *O*-glycans potentially enables sialic acid residues to modulate the adhesion of microorganisms, either positively or negatively. This should be further studied on a broader range of bacteria and on purified *O*-glycan structures.

In this study, we demonstrated that sialylated core 3 glycans are key factors in the binding of intestinal commensals, probiotics and pathogens to mucins. However, we also showed that microorganisms have the ability to bind, to a lesser extent, to gastric mucins, which are mainly composed of non-sialylated core 2 *O*-glycans. These results may be explained by the high expression of blood group antigens on gastric mucins. Indeed, several studies have reported the capacity of bacterial adhesins to recognize blood group determinants. For example, FedF, the adhesion on F18 fimbriae from shiga-secreting *E. coli*, binds to ABH type 1 and sulphated H type 2 blood group antigens [68]. Family 1 of solute-binding proteins from Bifidobacterium, which is part of the ABC transporters, has been identified as able to bind mucin *O*-glycans, human milk oligosaccharides and blood group antigens [69]. Blood group antigens are also expressed by mucins from the jejunum and ileum and may be recognized by microorganisms, thus at least partly explaining their binding.

In conclusion, we have developed a rapid and sensitive assay to evaluate the binding of microorganisms to mucins, with smaller amounts of material than in conventional mucin binding experiments. We have demonstrated its efficiency to study biologically relevant interactions between mucin glycotopes and bacteria. The use of human mucins rather than mucins derived from cell culture or from commercial sources is crucial to identify the exact oligosaccharide structures involved in bacteria–host crosstalk. This will help to clarify the molecular mechanisms of *O*-glycan mediated interactions in infectious diseases as well asselecting probiotics with a high capacity for mucus adhesion and colonization.
